# Automatic Detection of Cone Photoreceptors With Fully Convolutional Networks

**DOI:** 10.1167/tvst.8.6.10

**Published:** 2019-11-12

**Authors:** Jared Hamwood, David Alonso-Caneiro, Danuta M. Sampson, Michael J. Collins, Fred K. Chen

**Affiliations:** 1 School of Optometry & Vision Science, Queensland University of Technology, Queensland, Australia; 2 Centre for Ophthalmology and Visual Science (incorporating Lions Eye Institute), The University of Western Australia, Perth, Western Australia, Australia; 3 Surrey Biophotonics, Centre for Vision, Speech and Signal Processing and School of Biosciences and Medicine, The University of Surrey, Guildford, UK; 4 Department of Ophthalmology, Royal Perth Hospital, Perth, Western Australia, Australia

**Keywords:** deep learning, cone detection, image analysis, photoreceptors

## Abstract

**Purpose:**

To develop a fully automatic method, based on deep learning algorithms, for determining the locations of cone photoreceptors within adaptive optics scanning laser ophthalmoscope images and evaluate its performance against a dataset of manually segmented images.

**Methods:**

A fully convolutional network (FCN) based on U-Net architecture was used to generate prediction probability maps and then used a localization algorithm to reduce the prediction map to a collection of points. The proposed method was trained and tested on two publicly available datasets of different imaging modalities, with Dice overlap, false discovery rate, and true positive reported to assess performance.

**Results:**

The proposed method achieves a Dice coefficient of 0.989, true positive rate of 0.987, and false discovery rate of 0.009 on the first confocal dataset; and a Dice coefficient of 0.926, true positive rate of 0.909, and false discovery rate of 0.051 on the second split detector dataset. Results compare favorably with a previously proposed method, but this method provides quicker (25 times faster) evaluation performance.

**Conclusions:**

The proposed FCN-based method demonstrates that deep learning algorithms can achieve accurate cone localizations, almost comparable to a human expert, while labeling the images.

**Translational Relevance:**

Manual cone photoreceptor identification is a time-consuming task due to the large number of cones present within a single image; using the proposed FCN-based method could support the image analysis task, drastically reducing the need for manual assessment of the photoreceptor mosaic.

## Introduction

Cone photoreceptors are vital for human vision. Specifically, these cells serve daylight and color vision. Diseases such as Stargardt's disease,^1^ retinitis pigmentosa,^2^ choroideremia,^3^ and macular degeneration^4^ are characterized by the loss of photoreceptors leading to impaired vision. A way to image the photoreceptor array is using an adaptive optics scanning laser ophthalmoscope (AOSLO). Two common variants of the AOSLO imaging modality are confocal and split detector, each providing slightly different information on photoreceptor structure.^5^,^6^

Regardless of the method use to acquire the images, the cones must be located within the image to create quantifiable information and extract metrics, such as cone density and spacing and packing arrangements. Given the high density of cones within the image, manual cone identification can be time-consuming and inconsistent. Several automatic or semi-automatic methods have been proposed to create a faster and more consistent cone detection process. Some methods are based on standard image analysis techniques: image histogram analysis,^7^ multi-scale modelling and normalized cross-correlation,^8^ a circular Hough transform,^9^ and multiscale circular voting.^10^ In recent years, machine learning methods have also been applied to this problem. Cunefare et al.^11^ proposed a so-called “patch-based” method involving generating a probability map through a sliding window convolutional neural network (CNN) and then postprocessing this probability map to locate cone positions. The CNN, which works on a small window of the entire image, generates a binary (two-class) classification of the image as either the patch centered on a cone or not centered on a cone. By moving the window along different sections of the image, the probability map is obtained. The postprocessing method, which is needed to extract peaks from the probability map (cone locations), contains several steps and several tunable parameters. Heisler et al.^12^ investigated the use of transfer learning on the network of Cunefare et al.^11^ to enable classifications of previously unseen data collected from a different imaging modality (AO scanning laser ophthalmoscope). Davidson et al.^13^ proposed a method using a multidimensional Recurrent Neural Network (RNN), which generates a probability map for the entire image in a single set of computations.

Patch-based and CNNs have commonly been applied to ophthalmic medical images, such as retinal segmentation or classification, and provide state-of-the-art performance in these areas.^14^–^16^ Fully Convolutional Networks (FCNs) are an extension of CNNs.^17^ The main benefit of an FCN is the ability to process the entirety of an image at once and provide a per-pixel probability map. FCNs are commonly used for object segmentation, region labeling, or other per-pixel operations^17^ and have been used for geographic atrophy segmentation in retinal tomography images.^18^ FCNs have been commonly used in medical image processing for problems such as retinal layer segmentation,^19^ segmentation of neuronal structures,^20^ and cell detection.^21^ For per-pixel operations, FCNs are commonly quicker than a patch-based CNN or RNN, as the FCN only passes over the data once, whereas data are repeatedly evaluated in the case of a patch-based CNN and multiple recurrent loops increase the number of operations in the case of an RNN.^22^

In this work, we propose the application on a FCN for cone detection in confocal and split detector AOSLO images. We use a previously published method, based on a patch-based technique,^11^ as a baseline to assess the benefit that a FCN approach may have in this particular problem.

## Methods

The method for finding cones consists of two steps. The first step is the generation of a probability map through an FCN, and the second step is postprocessing the probability map to a collection of cone locations. Training of the neural network and parameter selection of the postprocessing are done separately. The FCN was trained on the given training images for the dataset (details below), for 50 epochs. After training was complete, the training images were processed through the FCN and then generated probability maps were used to optimize the detection parameters of the cone location postprocessing according to a parameter sweep. This trained combination of FCN and detection parameters was then used to segment the test images giving the final performance.

### Datasets

The FCN method was trained and evaluated on two publicly available datasets, namely, one consisting of confocal samples, acquired by Garrioch et al.,^5^ and one consisting of split detector samples acquired by Cunefare et al.^6^; data associated with this publication and used here for testing of the proposed methods were obtained online (https://github.com/DavidCunefare/CNN-Cone-Detection).

The confocal set consists a total of 840 images, split into a testing set of 640 images and a training set of 200 images. Full acquisition parameters are given in Garrioch.^5^ Given some images had different dimensions and this would complicate setting the network, all images were cropped to a common pixel size, using the central 144 × 144-pixel region, corresponding to an area ranging from 62 to 74 μm^2^. The split detector set consists of 264 images, split into a testing set of 80 images and a training set of 184 images. Full acquisition parameters are given in Cunefare et al.^6^ Like the confocal set, all images were cropped to a central 144 × 144-pixel region, corresponding to an area ranging from 56 to 68 μm^2^. Both sets were independently trained and evaluated, with independent networks and detection parameters.

### Preprocessing and Augmentation

**Figure 1:**
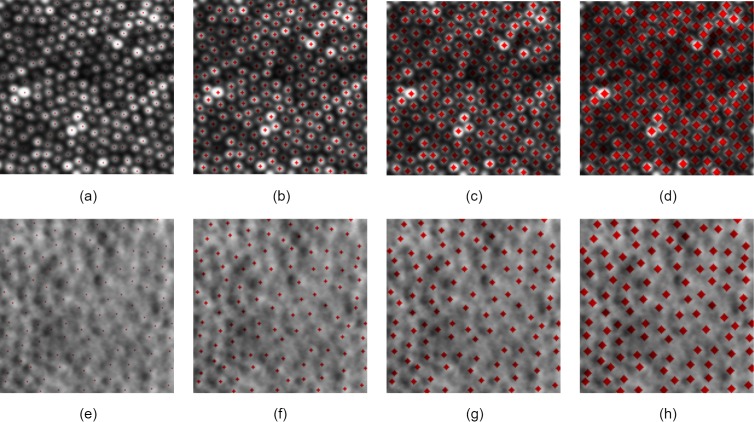
(a–d) Confocal image samples and (e–h) split detector image samples. (a, e) 0-pixel padding. (b, f) 1-pixel padding. (c, d) 2-pixel padding. (d, h) 3-pixel padding.

Given the relative sparseness of the pixels identified as being “cone location” in a single image, several modifications to create the ground truth maps (cone labels) were tested. A number of pixels from 0 to 3 were added in a diamond shape around each positive sample to give a larger number of positive samples to aid learning and assess if bias in the sample had an effect on performance. A size of 0 indicates a single pixel used as a mask, as shown in Figure 1.

Additionally, in the case of the confocal dataset, rotational augmentation in steps of 90 degrees was used because the confocal modality is rotation invariant. It is worth noting that split detector images are not rotation invariant due to the scan being created from the difference of two offset channels, and preliminary testing confirmed adding rotational augmentation to the training data reduced performance while testing on nonrotated images.

### Fully Convolutional Network

**Figure 2:**
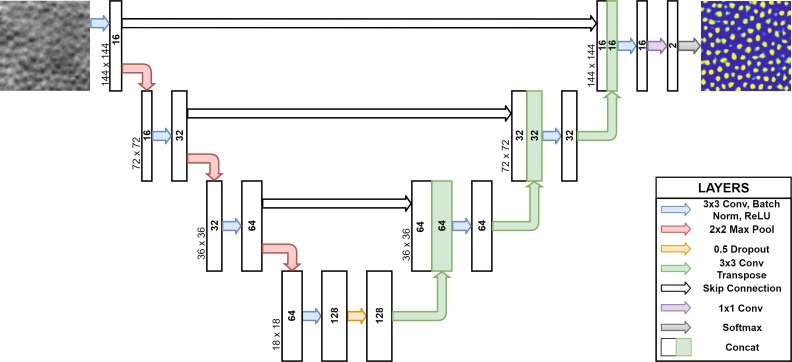
Proposed modified U-Net architecture.

The network used for this method was a modified U-net (Fig. 2) to have only a single convolutional-ReLU-batch norm block per pooling layer, zero padding to maintain input-output size parity, and a dropout of 0.5 at the bottleneck. All images used as inputs to the network were normalized on a per-image basis to be between 0 and 1 inclusive. A detailed explanation of the original network can be found elsewhere,^20^ for completeness; only a brief summary of the method is provided here. The network has a contracting path (encoder) followed by an expanding path (decoder), both of which contain a large number of feature channels giving the network greater capacity to propagate contextual information. To improve pixel-wise localization, “skips” were added to concatenate each feature map in the expanding path (decoder) with the corresponding feature map at the same level in the contracting path (encoder).^20^

Figure 2 shows the exact structure of the network. Convolutional layers are the main learnable operation within the network, where an input feature map is convolved by a filter of size W×H×F, where W indicates width, H indicates height, and F indicates the number of filters. Rectified linear units provide activations, only allowing an output if the input is above zero. Batch norm layers are used to normalize training, keeping inputs bounded between 0 and 1. Max pooling layers subsample the input feature map by taking the largest value within a W×H region every S pixels in either dimension. Dropout layers commonly improve generalization and prevent overfitting to the training data and randomly turn off individual input pixels in the feature map at rate P. Transposed convolutional layers increase the size of the input feature map by S with a kernel of size W×H×F. Concatenation layers join two or more other layers together by adding extra channels. Softmax layers output the relative probabilities for each channel in the input feature map.

### Cone Localization

The output from the FCN is a probability map of the same size as the input image, with a gradient from background prediction to cone prediction over a width of several pixels in practice. In order to reduce this probability map to a list of cones positions, the detection scheme used by Cunefare et al.^11^ was applied to the probability maps. This allows the direct comparison of the methods (patch-based versus FCN).

The first step of this method is smoothing the probability map with a Gaussian filter of standard deviation *σ*. The second step is extended-maxima transform to find maximal regions where the maximum intraregion height difference is less than H. The third step is to filter the maximal regions based on the original probability map against a minimal threshold T. Finally, the centroids of the remaining clusters are taken as the cone locations. The parameters *σ*, H, and T were tested over 0 to 2 in steps of 0.1, 0 to 1 in steps of 0.1, and 0 to 0.3 in steps of 0.05, followed by 0.4 and 0.5, respectively.

### Testing

To compare the performance of the proposed method to existing methods in the literature the true positive rate (TPR), false detection rate (FDR), and Dice overlap (Dice) are given for all methods. These are based on the number of true positives (*T_p_*), where both automatic methods and truth indicate a cone; false positives (*F_p_*), where the automatic methods indicate a cone, but there is no corresponding cone in the truth; and false negatives (*F_n_*), where a cone is indicated by truth, but is not predicted by an automatic method. The equations for these metrics are shown below in Equations 1.1 to 1.3.

\newcommand{\bialpha}{\boldsymbol{\alpha}}\newcommand{\bibeta}{\boldsymbol{\beta}}\newcommand{\bigamma}{\boldsymbol{\gamma}}\newcommand{\bidelta}{\boldsymbol{\delta}}\newcommand{\bivarepsilon}{\boldsymbol{\varepsilon}}\newcommand{\bizeta}{\boldsymbol{\zeta}}\newcommand{\bieta}{\boldsymbol{\eta}}\newcommand{\bitheta}{\boldsymbol{\theta}}\newcommand{\biiota}{\boldsymbol{\iota}}\newcommand{\bikappa}{\boldsymbol{\kappa}}\newcommand{\bilambda}{\boldsymbol{\lambda}}\newcommand{\bimu}{\boldsymbol{\mu}}\newcommand{\binu}{\boldsymbol{\nu}}\newcommand{\bixi}{\boldsymbol{\xi}}\newcommand{\biomicron}{\boldsymbol{\micron}}\newcommand{\bipi}{\boldsymbol{\pi}}\newcommand{\birho}{\boldsymbol{\rho}}\newcommand{\bisigma}{\boldsymbol{\sigma}}\newcommand{\bitau}{\boldsymbol{\tau}}\newcommand{\biupsilon}{\boldsymbol{\upsilon}}\newcommand{\biphi}{\boldsymbol{\phi}}\newcommand{\bichi}{\boldsymbol{\chi}}\newcommand{\bipsi}{\boldsymbol{\psi}}\newcommand{\biomega}{\boldsymbol{\omega}}\begin{equation}\tag{1}{\rm{TPR}} = {{{T_p}} \over {{T_p} + {F_n}}}\end{equation}

\newcommand{\bialpha}{\boldsymbol{\alpha}}\newcommand{\bibeta}{\boldsymbol{\beta}}\newcommand{\bigamma}{\boldsymbol{\gamma}}\newcommand{\bidelta}{\boldsymbol{\delta}}\newcommand{\bivarepsilon}{\boldsymbol{\varepsilon}}\newcommand{\bizeta}{\boldsymbol{\zeta}}\newcommand{\bieta}{\boldsymbol{\eta}}\newcommand{\bitheta}{\boldsymbol{\theta}}\newcommand{\biiota}{\boldsymbol{\iota}}\newcommand{\bikappa}{\boldsymbol{\kappa}}\newcommand{\bilambda}{\boldsymbol{\lambda}}\newcommand{\bimu}{\boldsymbol{\mu}}\newcommand{\binu}{\boldsymbol{\nu}}\newcommand{\bixi}{\boldsymbol{\xi}}\newcommand{\biomicron}{\boldsymbol{\micron}}\newcommand{\bipi}{\boldsymbol{\pi}}\newcommand{\birho}{\boldsymbol{\rho}}\newcommand{\bisigma}{\boldsymbol{\sigma}}\newcommand{\bitau}{\boldsymbol{\tau}}\newcommand{\biupsilon}{\boldsymbol{\upsilon}}\newcommand{\biphi}{\boldsymbol{\phi}}\newcommand{\bichi}{\boldsymbol{\chi}}\newcommand{\bipsi}{\boldsymbol{\psi}}\newcommand{\biomega}{\boldsymbol{\omega}}\begin{equation}\tag{2}{\rm{FDR}} = {{{F_p}} \over {{F_p} + {T_p}}}\end{equation}

\newcommand{\bialpha}{\boldsymbol{\alpha}}\newcommand{\bibeta}{\boldsymbol{\beta}}\newcommand{\bigamma}{\boldsymbol{\gamma}}\newcommand{\bidelta}{\boldsymbol{\delta}}\newcommand{\bivarepsilon}{\boldsymbol{\varepsilon}}\newcommand{\bizeta}{\boldsymbol{\zeta}}\newcommand{\bieta}{\boldsymbol{\eta}}\newcommand{\bitheta}{\boldsymbol{\theta}}\newcommand{\biiota}{\boldsymbol{\iota}}\newcommand{\bikappa}{\boldsymbol{\kappa}}\newcommand{\bilambda}{\boldsymbol{\lambda}}\newcommand{\bimu}{\boldsymbol{\mu}}\newcommand{\binu}{\boldsymbol{\nu}}\newcommand{\bixi}{\boldsymbol{\xi}}\newcommand{\biomicron}{\boldsymbol{\micron}}\newcommand{\bipi}{\boldsymbol{\pi}}\newcommand{\birho}{\boldsymbol{\rho}}\newcommand{\bisigma}{\boldsymbol{\sigma}}\newcommand{\bitau}{\boldsymbol{\tau}}\newcommand{\biupsilon}{\boldsymbol{\upsilon}}\newcommand{\biphi}{\boldsymbol{\phi}}\newcommand{\bichi}{\boldsymbol{\chi}}\newcommand{\bipsi}{\boldsymbol{\psi}}\newcommand{\biomega}{\boldsymbol{\omega}}\begin{equation}\tag{3}{\rm{Dice}} = {{2 \times {T_p}} \over {2 \times {T_p} + {F_p} + {F_n}}}\end{equation}

**Figure 3:**
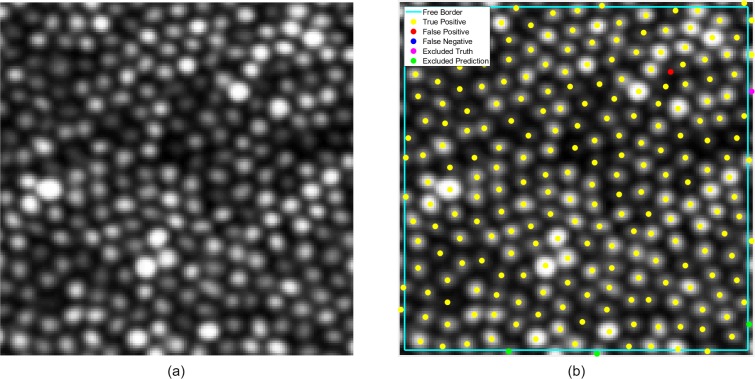
Visualization of cone matching and free zone. (a) raw image and (b) corresponding annotated version of the image. Yellow denotes true positives, red denotes false positives, and blue denotes a false negative (not present in this plot). The free zone is delineated by the cyan border. Inside this zone, magenta denotes excluded truths and green disregarded predictions.

Given the cropping of the original images and the possible confusion around borders where positive samples may lie outside the area of the image, a padding of 2 pixels around the inside of each border was added to a “free zone,” with the remainder of the image serving as a “testing area.” In the event that a prediction in the testing area had no corresponding true positive in the testing area but it did in the free zone, this was treated as a positive prediction. Positive samples in the free zone that had no corresponding prediction in the testing area were not treated as negatives (Fig. 3).

Patch-based methods can be trained on images of different sizes if a consistent size patch is extracted. However, to ensure a fair comparison between methods, the same central region used for the FCN was extracted from the full segmentation. To assess the effects of the different architectures on performance, a repeated measures analysis of variance was performed to examine the statistical significance of the different metrics associated with these factors.

## Results

All proposed methods were trained and tested in MATLAB 2018b (MathWorks, Natick, MA) by using the first party deep learning libraries, on a computer with a Xeon E5-2620v4 and an Nvidia Titan Xp. The patch-based networks^11^ were taken directly from an open source implementation for comparison purposes and were trained and tested on MATLAB 2018b with MatConvNet 1.0-beta25, on a computer with a Xeon E5-2620v4 and an Nvidia Titan Xp.

Regarding the computational time, the proposed FCN method generates a probability map for a 144 × 144-pixel image in 0.03 seconds, compared to 0.85 seconds for the patch-based CNN approach with an identical image. As both methods use the same cone location algorithm, both methods have the same running time added. The cone location step took 0.008 seconds on average, so the total for the FCN method is 0.038 seconds, and the total for the patch-based CNN is 0.858 seconds.

**Figure 4:**
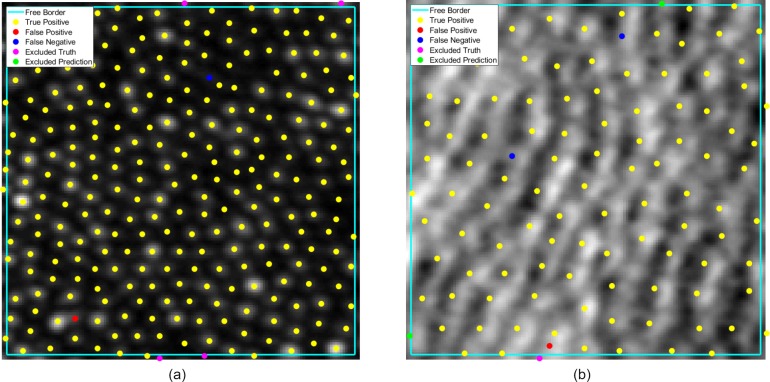
Visualization of model performance on (a) confocal image and (b) split detector image. Yellow denotes true positives, red denotes false positives, and blue denotes a false negative. The free zone is delineated by the cyan border. Inside this zone, magenta denotes excluded truths and green denotes disregarded predictions.

To assess the performance and the repeatability of the method, each network was trained from scratch and tested four times, with identical testing and training data sets and with results recorded independently, and the average of three metrics (and their standard deviation) across all networks is presented. No transfer learning is performed. Instead, each network is trained from scratch with weights initialized using small random values. Given the inherent randomness with neural networks, this serves to control for instabilities during initialization and training and gives a more accurate idea of what performance window is to be expected if training this network independently. Tables 1 and 2 summarize the performance on the confocal dataset and split detector dataset, respectively, and provide a comparison with existing methods. Figure 4 provides a visual comparison on the two different imaging modalities. In this work, we present the performance as the mean (and standard deviation) of the four runs to assess both performance and consistency of the network. The small standard deviation across the different tested conditions ensures the mean values provide a representative picture of performance.

**Table 1:** Mean and (Standard Deviation) Performance for the Confocal Dataset^a^

Method	True Positive Rate	False Discovery Rate	Dice Coefficient
Patch-based^11^	0.984 (0.014)	**0.007** (**0.011**)	0.988 (**0.010**)
FCN 0 wide	0.984 (0.016)	0.009 (0.014)	0.987 (0.013)
FCN 1 wide	0.984 (0.015)	0.008 (0.013)	0.987 (0.011)
FCN 2 wide	0.985 (0.015)	0.008 (0.012)	0.988 (0.011)
FCN 3 wide	**0.987** (**0.013**)	0.009 (0.013)	**0.989** (0.011)

a The best result for each performance metric is highlighted in bold text.

**Table 2:** Mean and (Standard Deviation) Performance for the Split Detector Dataset^a^

Method	True Positive Rate	False Discovery Rate	Dice Coefficient
Patch-based^11^	0.898 (0.095)	0.056 (0.062)	0.916 (0.065)
FCN 0 Wide	0.881 (0.083)	0.063 (0.069)	0.905 (0.065)
FCN 1 Wide	0.888 (0.078)	0.066 (0.065)	0.908 (0.055)
FCN 2 Wide	0.903 (**0.074**)	0.061 (0.060)	0.918 (0.055)
FCN 3 Wide	**0.909** (0.076)	**0.051** (**0.051**)	**0.926** (**0.052**)

a The best result for each performance metric is highlighted in bold text.

## Discussion

Across both datasets, the proposed FCN method with the three-wide padded data provides the best performance, in terms of the mean value of the different evaluated metrics. On comparison to the previously proposed patch-based technique,^11^ the margin between existing patch based and the FCN methods is greater in the split detector dataset where the three-wide FCN exhibited the best performance across the three measured parameters. However, none of the three parameters were statistically significantly different to the Cunefare (*P* > 0.01). For the confocal dataset, all three metrics were statistically significantly different to the Cunefare (*P* < 0.01); although the differences were small in magnitude, the FCN shows superior performance for Dice and True positive rate. The network was also evaluated against the multidimensional RNN proposed by Davidson and colleagues.^13^ However, because this particular network was designed to segment cones in the split detector image, the performance between datasets showed a big disparity and was not compared with the proposed method. The split detector had a good performance (true positive rate = 0.775, false discovery rate = 0.042, Dice = 0.849), whereas the confocal presented poor metrics (true positive rate = 0.233, false discovery rate = 0.007, Dice = 0.376). Changes in the network architecture may be needed to improve performance.

A limitation of the proposed method is the two-step approach. It is likely that better performance could be achieved by evaluating the FCN on the exact metric used for performance in the training stages. Finding a way to change the network to directly output a collection of cone positions would be ideal; however, this is not a trivial problem and should be considered in future studies.

Cone photoreceptor imaging modalities can have a small depth of focus, so blur can be present in the image.^23^ Understanding how the different models deal with this blur and the impact on performance should be considered in future studies. Also, future studies should evaluate the proposed method on datasets from pathological eyes, as many diseases can change the mosaic structure significantly,^1^–^4^ as well as with other imaging modalities such as dual split and confocal.^24^ Whether similar performance can be achieved with datasets from pathological eyes remains to be seen.

### Conclusion

FCNs have proven useful for a number of image analysis tasks. In this work, the proposed end-to-end FCN method is able to provide a fast detection of cones in two different image modalities. The overall performance of the method is comparable or superior to previously proposed methods that are patch-based but with the added advantage that it runs in a fraction of the time.

## References

[1] Chen Y, Ratnam K, Sundquist SM (2011). Cone photoreceptor abnormalities correlate with vision loss in patients with Stargardt disease. *Invest Ophthalmol Vis Sci*.

[2] Makiyama Y, Ooto S, Hangai M (2013). Macular cone abnormalities in retinitis pigmentosa with preserved central vision using adaptive optics scanning laser ophthalmoscopy. *PLoS One*.

[3] Morgan JIW, Han G, Klinman E (2014). High-resolution adaptive optics retinal imaging of cellular structure in choroideremiaadaptive optics imaging in choroideremia. *Invest Ophthalmol Vis Sci*.

[4] Qin J, Rinella N, Zhang Q (2018). OCT angiography and cone photoreceptor imaging in geographic atrophy. *Invest Ophthalmol Vis Sci*.

[5] Garrioch R, Langlo C, Dubis AM, Cooper RF, Dubra A, Carroll J (2012). Repeatability of in vivo parafoveal cone density and spacing measurements. *Optom Vis Sci*.

[6] Cunefare D, Cooper RF, Higgins B (2016). Automatic detection of cone photoreceptors in split detector adaptive optics scanning light ophthalmoscope images. *Biomed Opt Express*.

[7] Xue B, Choi SS, Doble N, Werner JS (2007). Photoreceptor counting and montaging of en-face retinal images from an adaptive optics fundus camera. *J Opt Soc Am A Opt Image Sci Vis*.

[8] Turpin A, Morrow P, Scotney B, Anderson R, Wolsley C (2011). Automated identification of photoreceptor cones using multi-scale modelling and normalized cross-correlation. *International Conference on Image Analysis and Processing*.

[9] Bukowska DM, Chew AL, Huynh E (2015). Semi-automated identification of cones in the human retina using circle Hough transform. *Biomed Opt Express*.

[10] Liu J, Jung H, Dubra A, Tam J (2017). Automated photoreceptor cell identification on nonconfocal adaptive optics images using multiscale circular voting. *Invest Ophthalmol Vis Sci*.

[11] Cunefare D, Fang L, Cooper RF, Dubra A, Carroll J, Farsiu S (2017). Open source software for automatic detection of cone photoreceptors in adaptive optics ophthalmoscopy using convolutional neural networks. *Sci Rep*.

[12] Heisler M, Ju MJ, Bhalla M (2018). Automated identification of cone photoreceptors in adaptive optics optical coherence tomography images using transfer learning. *Biomed Opt Express*.

[13] Davidson B, Kalitzeos A, Carroll J (2018). Automatic cone photoreceptor localisation in healthy and Stargardt afflicted retinas using deep learning. *Sci Rep*.

[14] Fang L, Cunefare D, Wang C, Guymer RH, Li S, Farsiu S (2017). Automatic segmentation of nine retinal layer boundaries in OCT images of non-exudative AMD patients using deep learning and graph search. *Biomed Opt Express*.

[15] Hamwood J, Alonso-Caneiro D, Read SA, Vincent SJ, Collins MJ (2018). Effect of patch size and network architecture on a convolutional neural network approach for automatic segmentation of OCT retinal layers. *Biomed Opt Express*.

[16] Lu W, Tong Y, Yu Y, Xing Y, Chen C, Shen Y (2018). Deep learning-based automated classification of multi-categorical abnormalities from optical coherence tomography images. *Transl Vis Sci Technol*.

[17] Long J, Shelhamer E, Darrell T (2015). Fully convolutional networks for semantic segmentation. *Proceedings of the IEEE Conference on Computer Vision and Pattern Recognition*.

[18] Ji Z, Chen Q, Niu S, Leng T, Rubin DL (2018). Beyond retinal layers: a deep voting model for automated geographic atrophy segmentation in SD-OCT images. *Transl Vis Sci Technol*.

[19] Roy AG, Conjeti S, Karri SPK (2017). ReLayNet: retinal layer and fluid segmentation of macular optical coherence tomography using fully convolutional networks. *Biomed Opt Express*.

[20] Ronneberger O, Fischer P, Brox T (2015). U-Net: convolutional networks for biomedical image segmentation. *International Conference on Medical Image Computing and Computer-Assisted Intervention*.

[21] Xie W, Noble J, Zisserman A (2018). Microscopy cell counting and detection with fully convolutional regression networks. *Computer Methods in Biomechanics and Biomedical Engineering: Imaging & Visualization*.

[22] Kugelman J, Alonso-Caneiro D, Read SA, Vincent SJ, Collins MJ (2018). Automatic segmentation of OCT retinal boundaries using recurrent neural networks and graph search. *Biomed Opt Express*.

[23] Alonso-Caneiro D, Sampson DM, Chew AL, Collins MJ, Chen FK (2018). Use of focus measure operators for characterization of flood illumination adaptive optics ophthalmoscopy image quality. *Biomed Opt Express*.

[24] Cunefare D, Langlo CS, Patterson EJ, Blau S, Dubra A, Carroll J, Farsiu S (2018). Deep learning based detection of cone photoreceptors with multimodal adaptive optics scanning light ophthalmoscope images of achromatopsia. *Biomed Opt Express*.

